# On the *Monacha* species of Lebanon (Gastropoda, Hygromiidae)

**DOI:** 10.3897/zookeys.311.5408

**Published:** 2013-06-20

**Authors:** Eike Neubert, Michel Bariche

**Affiliations:** 1 Naturhistorisches Museum der Burgergemeinde Bern, Bernastrasse 15, CH-3005 Bern, Switzerland; 2 Marine Biology & Ichthyology, Biology Dep., American University of Beirut

**Keywords:** Hygromiidae, *Monacha*, Lebanon, re-description

## Abstract

In this paper, all seven hitherto known species of the hygromiid genus *Monacha* from Lebanon are briefly characterised and illustrated, and distribution maps are supplied (*Monacha (Monacha) syriaca* (Ehrenberg, 1831), *Monacha (Monacha) nummus* (Ehrenberg, 1831), *Monacha (Monacha) obstructa* (L. Pfeiffer, 1842), *Monacha (Monacha) crispulata* (Mousson, 1861), *Monacha (Monacha) solitudinis* (Bourguignat, 1852), *Monacha (Monacha) bari* Forcart 1981, and *Monacha (Monacha)* cf. *compingtae* (Pallary, 1929)). One species, *Monacha (Monacha) bari* Forcart, 1981, is recorded for the country for the first time, and its relationship to *Monacha (Monacha) compingtae* (Pallary, 1929) is discussed. Based on recently collected specimens, the genital organs of a long time ignored species, *Helix solitudinis* Bourguignat, 1852 could be investigated. It is here re-described as a *Monacha* species endemic for Lebanon.

## Introduction

The malacofauna of Lebanon has been the focus of malacologists since quite early on. Among the earliest scientists collecting molluscs in this country, the famous German zoologists Friedrich Wilhem Hemprich and Christian Gottfried Ehrenberg from Berlin, who visited the area during their second voyage from 1821–1825, have to be mentioned. Their results were subsequently published by Ehrenberg (1828–1833). Since then, the country has been the target of many expeditions where zoological and/or botanical objects were collected. Already at that time, Beirut served as “headquarters” for collectors, and thus, nearby and easily accessible places like Wadi Beyrouth or Wadi Nahr el Kelb became type localities of dozens of species.

At the beginning of the 20^th^ century, a French expedition lead by Henri Gadeau de Kerville collected an enormous amount of continental molluscs from Syria in the wider sense, which were treated in two large volumes by Germain (1921). These specimens are housed in the MNHN in Paris. Later, the French malacologist Pallary, a keen connoisseur of the malacofauna of Northern Africa and home-based in Oran, Algeria, published some papers on the wider Palestinian area (Pallary 1929, 1939). Finally, the famous Lebanese zoologists Henriette and George Thomé presented a small comprehensive volume covering the terrestrial malacofauna of Lebanon (Thomé and Thomé 1988). Since then, no papers dealing exclusively with Lebanon have been published, and occurrences of continental molluscs have only been mentioned as by-products in papers having another scope. Quite recently, Bössneck (2011) reported on the results of his field trip to the country, where he (among others) mentioned two interesting species of *Monacha* from the Lebanon Mountains. These findings stimulated the present authors to amend these records with the results of their own research.

In Turkey, there is a large variety of species of the genus *Monacha*, where currently 54 species are known, with still several species remaining undescribed because of the low number of specimens available, or of lack of anatomical data (Hausdorf 2000). Nonetheless, the mountains stretching towards the south house a few more species of the genus, some of them are endemic, not well studied as of today, and have been only inadequately illustrated thus far. It is also our aim to raise awareness of this more or less ignored group of invertebrate animals living in Lebanon to local people, laymen as well as students.

## Material and methods

This paper summarises data obtained during two excursions by the authors in Lebanon in 2008 and 2011, and others by Bariche during the last years. Specimens were collected by hand, and stored in the respective institutional collections. The section “specimens examined” contains only information that has been personally confirmed by the authors, i.e. none of the records have been retrieved from the literature. The records of Boessneck have been verified by Hausdorf (and a few by Neubert), and have been added to the distribution data. Finally, it also includes all data related to the *Monacha* species from Lebanon, its vouchers can be found in the Pallary collection housed at the AUBM. All specimens were checked by Neubert during his visit at the AUBM in October 2011. Unfortunately, the whereabouts of the Thomé’s collection remains unclear. Inquiries at the MHNG, at the MNHN, and even personal contact of M. Bariche with the Thomés’ yielded no clarification. Thus, the voucher specimens listed in the book by Thomé and Thomé (1988) are not available at the moment.

All measurements in mm.

### Abbreviations for institutions

AUBMAmerican University of Beirut, mollusc collection, Lebanon.

MHNBNaturhistorisches Museum Basel, Switzerland.

MHNGMuséum d’histoire naturelle de la ville de Genève, Switzerland.

MNHNMuseum national d’histoire naturelle Paris, France

MZLMuséum d’Zoologie, Lausanne, Switzerland.

NMBENaturhistorisches Museum der Burgergemeinde Bern, Switzerland.

SMNSStaatliches Museum für Naturkunde Stuttgart, Germany

### Abbreviations for technical terms

Hshell height

Dshell diameter

PHperistome height

PDperistome diameter

## Results

### Familia Hygromiidae Tryon, 1866 Subfamilia Monachainae Wenz, 1930 (1904) Tribus Monachaini Wenz, 1930 (1904) Genus *Monacha (Monacha)* Fitzinger, 1833

#### Monacha (Monacha) syriaca

(Ehrenberg, 1831)

http://species-id.net/wiki/Monacha_syriaca

Figs 1 –3

Helix syriaca Ehrenberg, Symbolae Physicae: 7 [Berytum]. 1831

##### Specimens examined:

NMBE 506363/1, Hlaliye, 33.8393°N, 35.636°E, 475 m alt., 28.10.2011; NMBE 506362/2, Ajhbe, 34.0087°N, 35.7108°E, 1164 m alt., 27.10.2011; NMBE 506361/5, Aaramoun, 34.0153°N, 35.6973°E, 760 m alt., 27.10.2011; NMBE 506360/7, above Kfour, 34.0317°N, 35.6988°E, 895 m alt., 27.10.2011; NMBE 506359/3, Kfour, 34.0348°N, 35.6952°E, 817 m alt., 27.10.2011; NMBE 506358/2, Adma, 34.0166°N, 35.6489°E, 85 m alt., 27.10.2011; NMBE 505184/1, above Kfour, 34.0317°N, 35.6988°E, 895 m alt., 27.10.2011 (preserved); NMBE 25914/3, Trablous (= Tripoli), 34.43°N, 35.85°E, coll. Shuttleworth ex Verreaux; NMBE 508014/20, road between Ain Et Tine and Machgara, 33.516°N, 35.644°E, 1080 m alt., 21.08.2008; NMBE 508013/2, Jezzine, Mazrad el Mathané, 33.576°N, 35.519°E, 450 m alt., 21.08.2008; NMBE 508012/2, Nahr Bisri, 33.58°N, 35.535°E, 400 m alt., 21.08.2008; NMBE 508011/64, Jezzine, W of village, steep slope, 33.546°N, 35.575°E, 940 m alt., 21.08.2008; NMBE 508010/15, Nahr Ibrahim, Chouene, trail Chouene–Chouwan Lake, 34.081°N, 35.785°E, 450 m alt., 20.08.2008; NMBE 508009/6, Nahr Ibrahim, close to the estuary, 34.065°N, 35.643°E, 20.08.2008; NMBE 508008/1, Qanater al Zbeideh, Nahr Beirut, 33.85°N, 35.556°E, 80 m alt., 19.08.2008; NMBE 508007/10, Byblos, 34.122°N, 35.652°E, 20 m alt., 19.08.2008; NMBE 508006/72, Nahr Abu Ali close to Seraad, 34.382°N, 35.93°E, 630 m alt., 19.08.2008; NMBE 508005/3, Tourza, 34.281°N, 35.89°E, 870 m alt., 19.08.2008; NMBE 508004/32, Jazire, Nahr al Qasimiyah (part of the Litani), 33.32°N, 35.288°E, 15 m alt., 18.08.2008; NMBE 508003/2, Nahr al Damour, 33.704°N, 35.452°E, 20 m alt., 18.08.2008; NMBE 508002/1, Nahr Kfar Matta at Jisr el Kadi, 33.721°N, 35.558°E, 260 m alt., 17.08.2008; NMBE 508001/75, Ain al Jdeidi, 33.809°N, 35.63°E, 918 m alt., 17.08.2008; NMBE 508000/1, Nahr el Kalb, upper reach of Nahr al-Kalb near Daraya, 33.955°N, 35.706°E, 700 m alt., 16.08.2008; NMBE 507999/7, Nahr el Kalb, Jeita Grotto, cave entrance, 33.944°N, 35.641°E, 90 m alt., 16.08.2008; NMBE 507998/2, Nahr el Kelb, road to Jeita Grotto, limestone slope, 33.947°N, 35.637°E, 115 m alt., 16.08.2008; AUBM-MOLL0470/5, Ghazir, 34.0302°N, 35.6701°E, 326 m alt., 25.10.2009; AUBM-MOLL0473/13, Habboub, 34.1284°N, 35.686°E, 420 m alt., 11.10.2009; AUBM-MOLL0476/7, Jeita, 33.9448°N, 35.6387°E, 88 m alt., 26.09.2009; AUBM-MOLL0478/1, Nahr el Kelb, 33.9547°N, 35.5975°E, 30 m alt., 26.09.2009; AUBM-MOLL0481/1, Hamat, 34.2967°N, 35.6768°E, 100 m alt., 16.01.2010; AUBM-MOLL0482/4, Kfar Kouass, 34.1222°N, 35.6969°E, 630 m alt., 11.10.2009; AUBM-MOLL0485/3, Moultaa el Nahreyn, 33.7029°N, 35.4673°E, 110 m alt., 04.10.2009; AUBM-MOLL0487/5, Bteghrine, 33.9253°N, 35.7745°E, 1000 m alt., 07.10.2009; AUBM-MOLL0329/16, Jounieh, 33.3896°N, 35.6323°E, coll. Pallary; AUBM-MOLL0335/38, Tartous, 34.8905°N, 35.8707°E, coll. Pallary; AUBM-MOLL0337/40, Beirut, 33.8526°N, 35.4296°E, coll. Pallary; AUBM-MOLL0338/25, Tripoli, 34.4314°N, 35.8184°E, coll. Pallary.

##### Diagnosis.

shell depressed, with two white spiral bands, malleate teleoconch sculpture, aperture reinforced by a strong white lip, apertural rim deep red, umbilicus closed by columellar callus.

##### Description.

shell depressed, with an elevated broad conical spire, basic shell colour deep brown to yellowish brown, usually with a white subsutural band and a second white band at the shell’s periphery; two protoconch whorls, smooth; teleoconch with a malleate sculpture (i.e. looks like markings of a small hammer) and very fine radial growth lines, surface with a glossy shine; last whorl rounded to slightly compressed forming a blunt shoulder; last whorl abruptly bent towards the aperture; aperture oval and depressed, reinforced by a strong white lip; apertural rim sharply bounded, usually deep red to brownish; umbilicus always closed by a small columellar callus.

**Figures 1–2.**
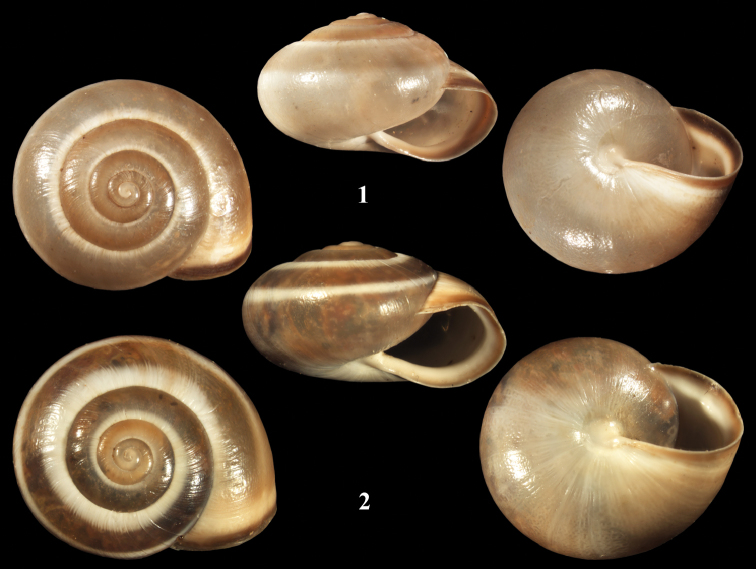
*Monacha (Monacha) syriaca*. **1** NMBE 508006, Nahr Abu Ali close to Seraad, D = 10.12 mm. **2** NMBE 508001, Ain al Jdeidi, D = 11.12 mm. – ×4, phot. E. Bochud.

**Measurements** (figured specimens). Fig. 1: H = 6.26; D = 10.12; PH = 3.17; PD = 5.38. Fig. 2: H = 6.18; D = 11.12; PH = 3.59; PD = 6.11.

**Figure 3.**
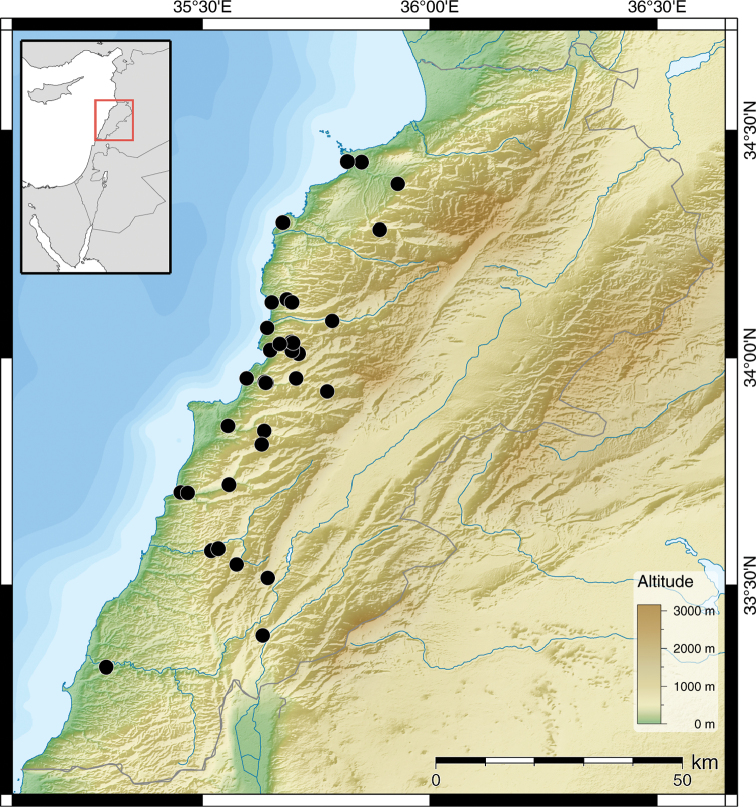
Distribution of *Monacha (Monacha) syriaca*.

##### Distribution.

This species is found widespread along the coastal areas in Lebanon. It usually lives at lower altitudes, but can occasionally be found up to 1000 m in more sheltered areas such as in the bottom of deep valleys.

##### Remarks.

This species cannot be mistaken for any other species in the area due to its characteristic colour pattern, teleoconch sculpture, and closed umbilicus.

#### Monacha (Monacha) nummus

(Ehrenberg, 1831)

http://species-id.net/wiki/Monacha_nummus

Figs 4, 5 , 8

Caracolla nummus Ehrenberg, Symbolae Physicae: 8 [Syrien]. 1831
Helix oxygyra Charpentier, Zeitschrift für Malakozoologie, 4: 131 [Bei Nahr und Kelb [sic!], zwei Lieues nördlich von Beirut, an Felsen]. 1847

##### Type specimens.

*oxygyra*: syntype in MZL.

##### Specimens examined.

NMBE 508022/6, Nahr Ibrahim, Chouene, trail Chouene–Chouwan Lake, 34.081°N, 35.785°E, 450 m alt., 20.08.2008; NMBE 508021/2, Nahr Ibrahim, at the road between Chouaya and Yahchouch, 34.064°N, 35.728°E, 780 m alt., 20.08.2008; NMBE 508020/3, Nahr Ibrahim, 34.079°N, 35.679°E, 80 m alt., 20.08.2008; NMBE 508018/1, Qanater al Zbeideh, Nahr Beirut, 33.85°N, 35.556°E, 80 m alt., 19.08.2008; NMBE 508017/5, Nahr Abu Ali close to Seraad, 34.382°N, 35.93°E, 630 m alt., 19.08.2008; NMBE 508016/20, Nahr el Kelb, Jeita Grotto, cave entrance, 33.944°N, 35.641°E, 90 m alt., 16.08.2008; NMBE 508015/4, Nahr el Kelb, road to Jeita Grotto, limestone slope, 33.947°N, 35.637°E, 115 m alt., 16.08.2008; NMBE 506357/1, Wadi Abadieh, 33.8434°N, 35.6344°E, 310 m alt., 28.10.2011; NMBE 506356/4, Kfar Hbab, 34.006°N, 35.6552°E, 113 m alt., 27.10.2011; NMBE 506355/3, Kfour, 34.0348°N, 35.6952°E, 817 m alt., 27.10.2011; NMBE 506354/12, Adma, 34.0166°N, 35.6489°E, 85 m alt., 27.10.2011; NMBE 25953/5, Beirut, 33.8526°N, 35.4296°E, coll. A. Vogt ex Bohny; AUBM-MOLL0466/9, AUBM-MOLL0478/1, AUBM-MOLL0480/13, Nahr el Kelb, 33.9547°N, 35.5975°E, 30 m alt., 26.09.2009; AUBM-MOLL0468/8, AUBM-MOLL0470/2, AUBM-MOLL0489/1, Ghazir, 34.0302°N, 35.6701°E, 326 m alt., 25.10.2009; AUBM-MOLL0476/5, Jeita, 33.9448°N, 35.6387°E, 88 m alt., 26.09.2009; AUBM-MOLL0552/2, Nahr Ibrahim, 34.0824°N, 35.802°E, 780 m alt.; AUBM-MOLL0343/10, Nahr el Kelb, coll. Pallary; AUBM-MOLL0344/19, Tripoli, 34.4314°N, 35.8184°E, coll. Pallary; AUBM-MOLL0345/9, Jounieh, 33.3896°N, 35.6323°E, coll. Pallary.

##### Diagnosis.

shell large, biconvex, periphery with a sharply bounded keel, umbilicus narrowly opened, broad periomphalum.

##### Description.

shell large and flat, biconvex, shell colour grey to brown; protoconch consisting of two smooth whorls; teleoconch whorls with a dense pattern of white ribs, that extend to the glossy underside of the shell; suture marked by a white sutural thread; ca. six regularly increasing whorls, periphery with a sharply bounded crimped keel, sometimes spire stepped because of whorls attaching somewhat below the keel during shell growth; last whorl not descending; aperture obliquely oval with an acute palatal edge, sometimes with a faint white lip; peristomial rim sharply bounded and simple; umbilicus always open, narrow, with a broad periomphalum.

**Measurements.** Syntype *oxygyra*: H = 5.91; D = 15.55; PH = 3.73; PD = 8.84. Fig. 5: H = 6.53; D = 16.15; PH = 3.39; PD = 8.67.

**Figures 4–7.**
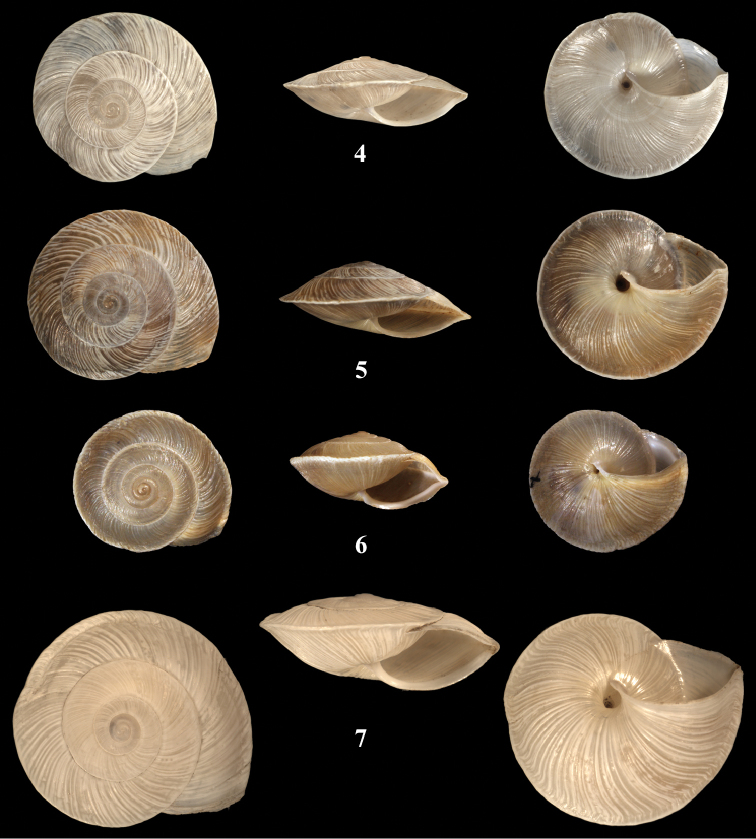
*Monacha* spp. **4** *Monacha (Monacha) nummus*, syntype *Helix oxygyra* Charpentier 1847, D = 15.55 mm **5** *Monacha (Monacha) nummus*, NMBE 508105, Beirut, Nahr el Kalb, Jeita Grotto, limestone rocks above entrance area to the cave, D = 16.15 mm **6** *Monacha (Monacha) spiroxia* (Bourguignat, 1868), lectotype MHNG 15378, Turkey, Alexandrette (= Iskenderun), D = 13.4 mm **7** *Monacha (Monacha) carinata* Hausdorf, 2000, holotype SMNS 50426, Turkey, Iskenderun, canyon of Sariseki, Iskenderun, D = 20.2 mm. – ×2, phot. Neubert, Richling.

**Figure 8.**
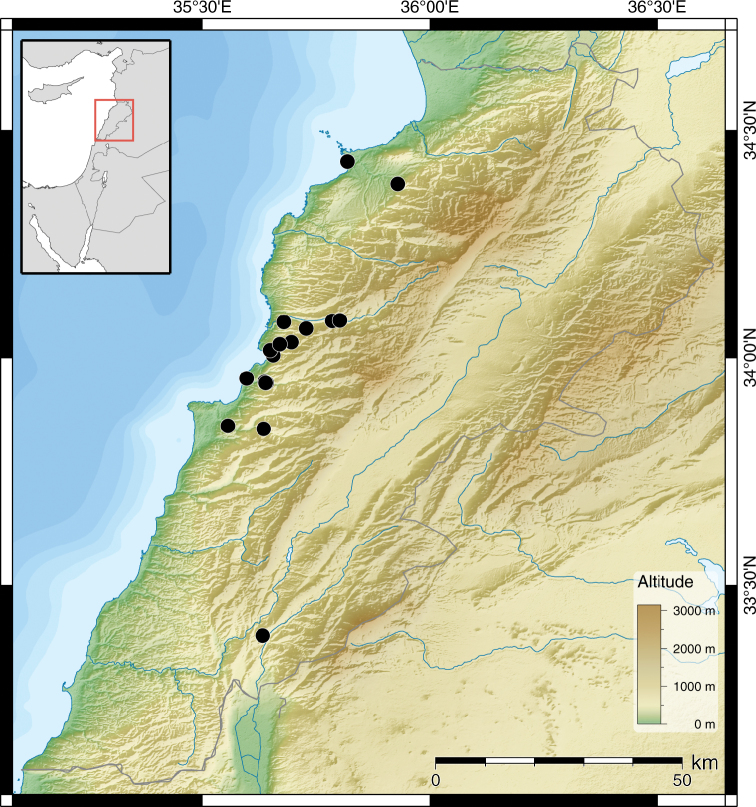
Distribution of *Monacha (Monacha) nummus*.

##### Distribution.

This species seems to be restricted to coastal regions in Lebanon.

##### Remarks.

This species can easily be confused with other keeled species known from the Levante area. To illustrate the differences, shells of *Monacha (Monacha) spiroxia* (Bourguignat, 1868) (Fig. 6) and *Monacha (Monacha) carinata* Hausdorf, 2000 (Fig. 7) are given. They differ in that the last has a larger shell with a more flat spire and a less pronounced keel. So far, these two species have only been recorded from the Hatay province of Turkey, while *Monacha (Monacha) nummus* seems to be restricted to Lebanon. *Monacha (Monacha) spiroxia* differs from *Monacha (Monacha) nummus* by being smaller, having a white lip in the aperture, a much narrower umbilicus and a teleoconch sculpture of small granules.

#### Monacha (Monacha) obstructa

(L. Pfeiffer, 1842)

http://species-id.net/wiki/Monacha_obstructa

Figs 9, 10

Helix obstructa L. Pfeiffer, Symbolae ad Historiam Heliceorum II: 35 [Tripoli]. 1842

##### Specimens examined.

NMBE 508032/12, Jazire, Nahr al Qasimiyah (part of the Litani), 33.32°N, 35.288°E, 15 m alt., 18.08.2008; NMBE 508031/32, Nahr al Damour, 33.704°N, 35.452°E, 20 m alt., 18.08.2008; NMBE 508030/7, Nahr el Kalb, Jeita Grotto, cave entrance, 33.944°N, 35.641°E, 90 m alt., 16.08.2008; AUBM-MOLL0492/1, Campus of the American University Beirut, 33.9016°N, 35.4781°E, 26.09.2009.

##### Diagnosis.

shell small, cream white, aperture with thick white labial callus, umbilicus closed, forming a characteristic funnel-shaped “pseudo-umbilicus”.

##### Description.

shell medium sized, spire broadly conical and somewhat elevated; protoconch consisting of 1.5 smooth whorls; teleoconch cream white, with faint riblets and a malleate sculpture (compare to *Monacha syriaca*), and evenly rounded whorls; last whorl slightly bending towards the aperture; aperture broadly oval, reinforced by a thick white labial callus, well discernible from the outside as a thick white callosity; peristomial rim sharply bounded, red; umbilicus closed, but last whorl forming a characteristic funnel-shaped “pseudo-umbilicus”.

**Figures 9, 10.**
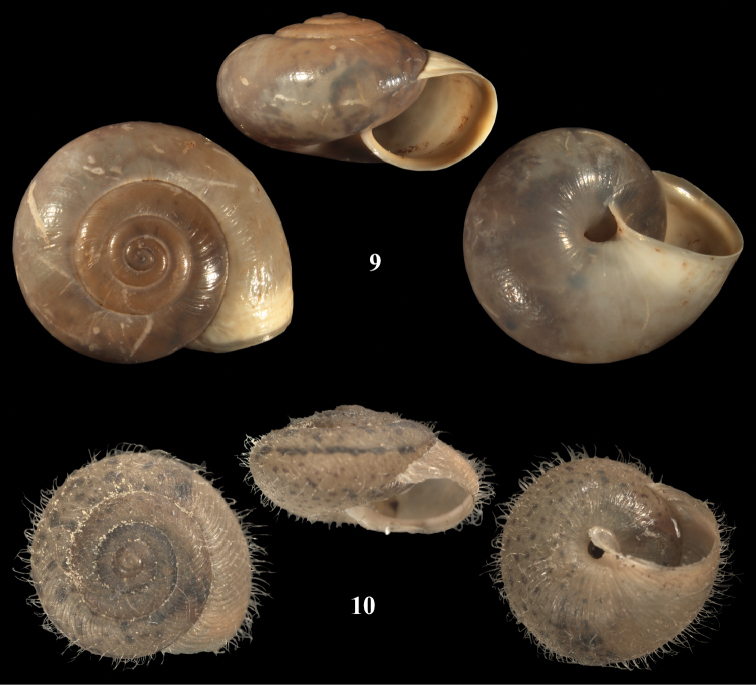
*Monacha* spp. **9** *Monacha (Monacha) obstructa*. NMBE 508031, Nahr al Damour, D = 11.83 mm **10** *Monacha (Monacha) crispulata*, Jordan, Ajlune Castle, 25.06.2007, leg. Z. Amr, D = 8.3 mm. – ×4, phot. E. Bochud.

**Measurements** (Fig. 9): H = 6.68; D = 11.83; PH = 4.2; PD = 5.85.

##### Distribution.

In Lebanon this species seems to be quite restricted, probably because it is confined to more arid or steppe-like areas like central Syria, where it is one of the most abundant snail species. These environmental conditions are not present in the western part of Lebanon with its humid and steep mountain slopes. However, it is the most widespread species within *Monacha*. Its distribution ranges from Egypt to Pakistan, and Turkey to Saudi Arabia (Neubert 1998), probably because it can be easily distributed by human activities.

##### Remarks.

This species is unmistakable for its funnel-shaped “pseudo-umbilicus”. Subadult shells can be differentiated from *Monacha syriaca* by the uniformity of its cream white shell.

#### Monacha (Monacha) crispulata

(Mousson, 1861)

http://species-id.net/wiki/Monacha_crispulata

Figs 10 , 11

Helix crispulata Mousson, Vierteljahresschrift der Naturforschenden Gesellschaft Zürich 6: 12 [Jerusalem ex Roth]. 1861

##### Specimens examined.

NMBE 515473/1, Nahr al Damour, 33.704°N, 35.452°E, 20 m alt., 18.08.2008; NMBE 515474/1, Qanater al Zbeideh, Nahr Beirut, 33.85°N, 35.556°E, 80 m alt., 19.08.2008.

##### Diagnosis.

shell small, teleoconch whorls with strong axial ribs, granules and long hairs.

##### Description.

shell small, spire slightly elevated; protoconch consists of 1.5 smooth whorls; teleoconch greyish to brownish, with strong axial ribs, whorls covered by a dense sculpture of small granules and very long, soft hairs; hair scars visible even in eroded shells; suture moderately deep; last whorl slightly bending towards the aperture; aperture broadly oval, reinforced by a white lip (discernible from the outside); peristomial rim sharply bounded; umbilicus open, partly covered by a triangular columellar callus.

**Figure 11.**
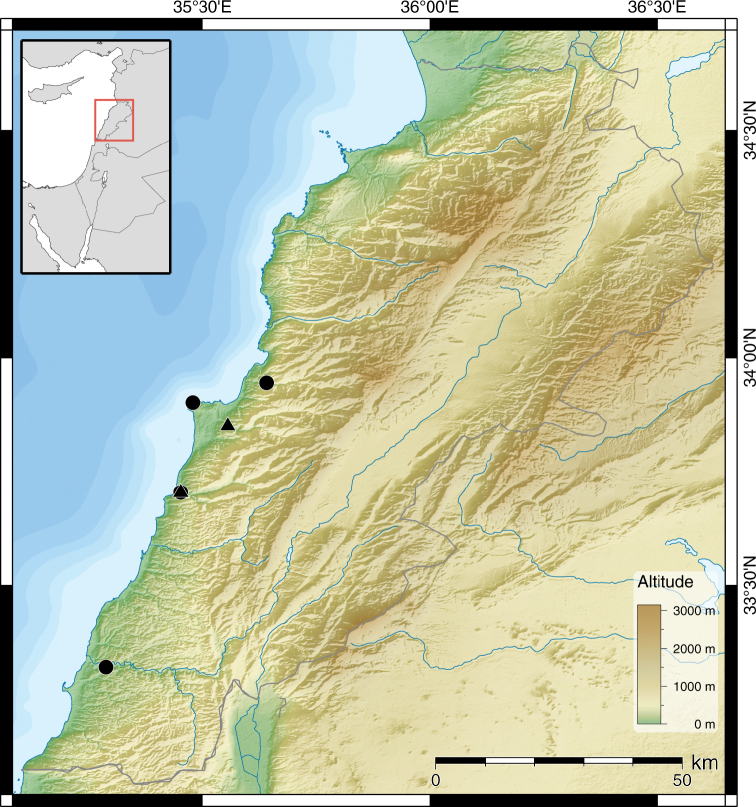
Distribution of *Monacha (Monacha) obstructa* (●) and *Monacha (Monacha) crispulata* (▲).

**Measurements** (Fig. 11): H = 4.72; D = 8.3; PH = 2.08; PD =4.76.

##### Distribution.

This seems to be a rare species in Lebanon. It has probably been overlooked because of its small size, or even misidentified, because its hairs can easily fall off, and the remaining shell is less characteristic. It is possible that this species is more widespread.

##### Remarks.

This species differs from all Lebanese *Monacha* species by its haired shell. Even eroded shells can be identified when using higher magnifications to see the axial ribs in combination with the granulated surface and remaining hair scars.

#### Monacha (Monacha) solitudinis

(Bourguignat, 1852)

http://species-id.net/wiki/Monacha_solitudinis

Figs 12 –14, 17

Helix solitudinis Bourguignat, Testacea novissimae…: 15 [Circa Heliopolim Syriae (Baalbek) habitat]. 1852
Helix solitudinis , – L. Pfeiffer, Monographia heliceorum viventium IV: 280. 1859
Helix solitudinis , – L. Pfeiffer, Monographia heliceorum viventium V: 365. 1868
Helicella (Monacha) solitudinis , – Gude, Journal of Malacology, 9: 128. 1902
Helix (Monacha) solitudinis – Germain, Mollusques terrestres et fluviatiles de Syrie I: 156. 1921
Metafruticicola solitudinis , – Pallary, Mémoires présentés a l’Institut d’Ègypte 39: 8. 1939
Monacha sp., – Boessneck, Zoology in the Middle East, 54: figs 5a, 42, 48. 2011

##### Type specimens.

Figured syntype NHMG 16032a; second syntype NHMG 16032b; Baalbek 34.0061°N, 36.2122°E.

##### Specimens examined.

Bcharre, Jabal el Mekmel ca. 7 km SE of Bcharre, loose calcareous vegetation, 34.2138°N, 36.0683°E, 2600 m alt, leg. U. Boessneck et al.; NMBE 508041/1, NMBE 508044/12, AUBM-MOLL, Falougha above Soha water plant, 33.835°N, 35.756°E, 1610 m alt., 17.08.2008; NMBE 508043/3, Falougha next to Soha water plant, 33.835°N, 35.752°E, 1474 m alt., 17.08.2008; NMBE 508042/8, Falougha, 33.8352°N, 35.7561°E, 1591 m alt., 29.10.2011; NMBE 515472/1, ditto (preserved).

##### Diagnosis.

shell medium sized, with a white spiral band at the periphery of the last whorl, aperture subrectangular, umbilicus open, cylindrical, penial papilla large and stout, flagellum longer than epiphallus.

##### Re-description.

shell medium sized, spire depressed; protoconch consists of 2 smooth whorls; shell colour light brown to yellowish brown with a white spiral band at the periphery of the last whorl; teleoconch with a malleate sculpture and fine axial riblets; suture deep, simple; teleoconch with up to seven densely coiled whorls; last whorl slightly bending towards the aperture; aperture broadly subrectangular, reinforced by a small white lip; peristomial rim sharply bounded, simple; umbilicus open, cylindrical, a triangular columellar callus is indicated.

**Figure 12.**
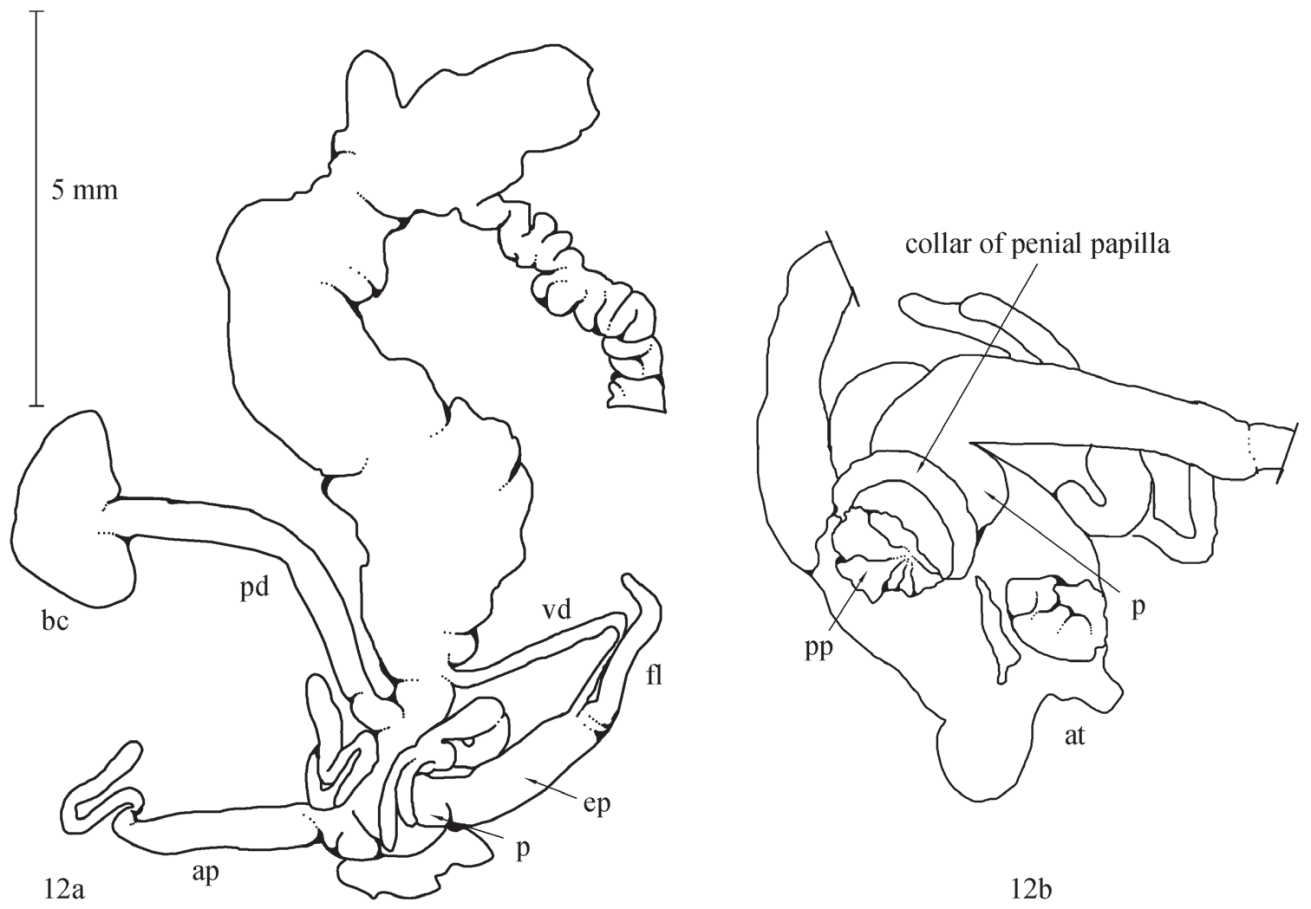
Anatomical drawing *Monacha (Monacha) solitudinis* **a** NMBE 515472, situs of genital organs, total length 10.9 mm **b** detail of atrium and penial papilla, not to scale. Abbreviations used: ap = appendicula; at = atrium; bc = bursa copulatrix; ep = epiphallus; fl = flagellum; ped = pedunculus; pp = penial papilla; vd = vas deferens.

Genital organs (Figs 12a, b): The only specimen that could be investigated was quite strongly contracted; it is a species of *Monacha* sensu str., because the genitalia show an appendicula, but the penial retractor muscle is missing. Penis very short (0.8 mm), penial papilla large, stout with a central perforation completely filling the atrial lumen (preservation artefact?), basis of the papilla with a strong collar (Fig. 12b); epiphallus a thick-walled tube, its lumen filled with several finely crenulated pilasters (1.9 mm); flagellum short (2.1 mm), but surmounting the length of the epiphallus; appendicula (4.9 mm) branching off the atrium, subdivided in a thicker basal part, and a shorter part with a narrower lumen; two glandulae mucosae poorly ramified, inserting in the middle of the vagina; vesicle of bursa copulatrix large, hammer-like, pedunculus quite long (4.7 mm); atrial lumen with two pilasters, one of them large, knob-like, the other narrow and elongate; right ommatophoran retractor passes left to the genital organs.

**Measurements.** Syntype figured: H = 9.2; D = 14.6; PH = 4.81; PD = 7.92. Fig. 14: H = 7.28; D = 13.31; PH = 4.34; PD = 7.32.

**Figures 13–17.**
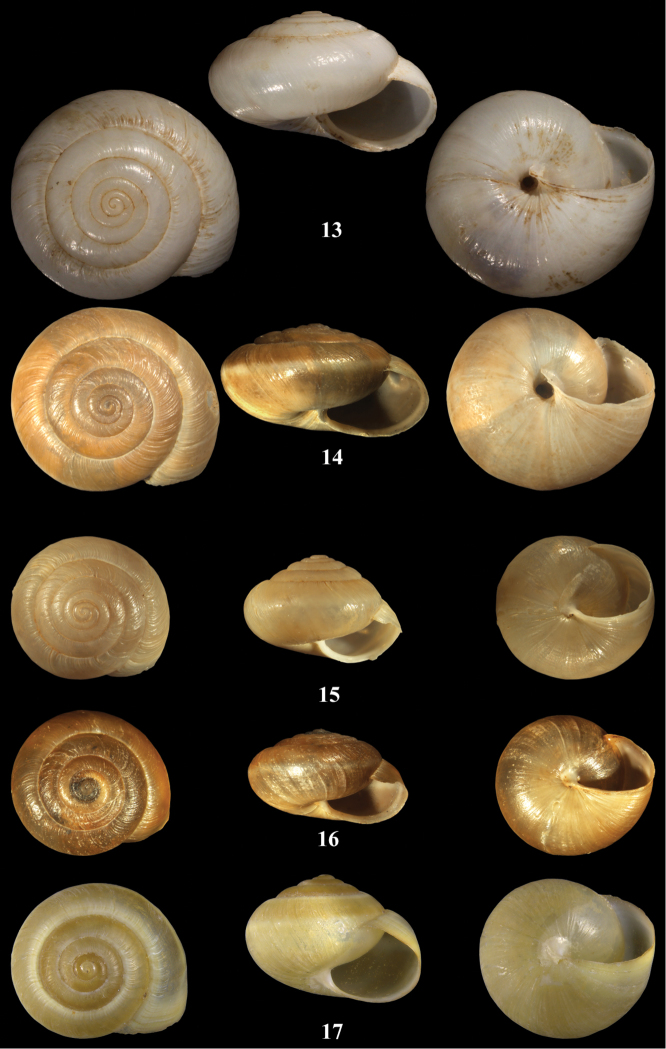
*Monacha* spp. **13** *Monacha (Monacha) solitudinis*. 13 syntype NHMG 16032a, Baalbek, D = 14.6 mm **14** NMBE 508041, Falougha above Soha water plant, D = 13.31 mm **15** *Monacha (Monacha) bari*, paratype NHMB 11167a, Israel, Mt. Hermon, D = 10.13 mm **16** *Monacha (Monacha) bari*, NMBE 508040, below Barouk Cedar Forest, D = 10.19 mm **17** *Monacha (Monacha)* cf. *compingtae*, Aammiq, D = XX mm. – ×4, phot. E. Bochud.

##### Distribution.

This species has only been recorded from two localities in Lebanon so far. The type locality Baalbek is not too far away and they were probably collected on the way towards Baalbek. Both new localities are in the central chain of Mt. Lebanon at high altitudes. The habitats are characterised by coarse limestone boulders with interspersed subalpine grassland vegetation and is covered by snow during winter. In October 2011, two living animals were found actively crawling over the rocks (ca. 10°C and heavy rainfall), while in August 2008, only dead shells were encountered.

##### Differential diagnosis.

This species differs from all other species in the Levante area by the shape of its umbilicus, which is cylindrical like a borehole. All other *Monacha* species with an open umbilicus deeply differ by either having a keeled (*Monacha nummus*) or a smaller and hairy shell (*Monacha crispulata*). *Monacha bari* and *Monacha compingtae* are conchologically similar to *Metafruticicola solitudinis*, but these species have a narrow and almost closed umbilicus (refer to the discussion of *Monacha bari*). Information on the genital organs of the two latter species is scarce, only Hausdorf (2000: 84) reported on a subadult specimen of *Monacha compingtae* from southern Turkey. In this species, the penial papilla is short (large but stout in *Metafruticicola solitudinis*), the epiphallus is longer than the flagellum (vice versa in *Metafruticicola solitudinis*), and the appendicula is much shorter than in *Metafruticicola solitudinis*. The specimen figured by Boessneck (2011) was re-investigated here; it matches the type specimen from Baalbek quite well, it is somewhat larger than the specimens from Falougha.

##### Remark.

The correct generic affiliation of this species remained unclear until now. In his original description, Bourguignat compared it to his *Helix camelina*, which is a species of the Oxychilidae; this text was uncritically copied by Pfeiffer (1859). Gude (1902) was the first to list it under the genus *Monacha*, but without any justification for doing so. Germain (1921), who remarked that this affiliation needs to be corroborated by an investigation of the genital organs, shared this point of view. Later, Pallary (1939) studied the type specimen and concluded that it should be classified within the genus *Metafruticiola*.

#### Monacha (Monacha) bari

Forcart, 1981

http://species-id.net/wiki/Monacha_bari

Figs 15, 16 , 18

Monacha (Monacha) bari Forcart, Basteria, 45: 105, fig. 11a–d [Hermon, Abstieg von der unteren Skiliftstation zum Nahal Arar (= Wadi Assal) 1600–1400 m ü.M. (etwa 33°15'N, 35°15'E). 1981

##### Type specimens.

*bari*: paratype MHNB 11167a.

##### Specimens examined.

NMBE 508040/11, below Barouk Cedar Forest, 33.7042°N, 35.6997°E, 1310 m alt., 29.10.2011; NMBE 508039/4, Qaroun Lake, 33.551°N, 35.682°E, 870 m alt., 21.08.2008.

##### Diagnosis.

shell small, spire slightly elevated, teleoconch has a faint white spiral band and fine axial riblets, umbilicus narrow closed by columellar callus.

##### Description.

shell small, spire conical, slightly elevated; protoconch consists of 1.5 smooth whorls; shell colour light brown to reddish brown, sometimes with a faint white spiral band at the periphery of the last whorl; teleoconch with fine axial riblets and a pattern of fine spiral threads (only visible under higher magnification); suture deep, simple; teleoconch with up to seven tightly coiled whorls; last whorl slightly bending towards the aperture; aperture broadly oval, reinforced by a white lip; peristomial rim sharply bounded, simple; umbilicus narrow, closed by a triangular columellar callus.

**Measurements.** Paratype *Monacha bari* figured: H = 6.9; D = 10.13; PH = 2.83; PD = 5.5. Fig. 16: H = 6.05; D = 10.19; PH = 2.94; PD =6.05.

**Figure 18.**
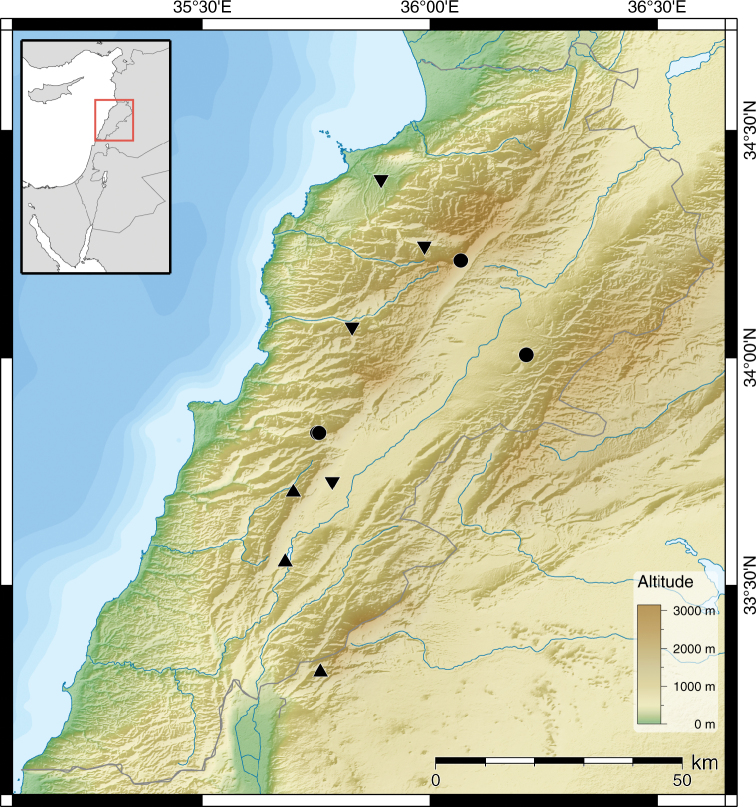
Distribution of *Monacha (Monacha) solitudinis* (●), *Monacha (Monacha) bari* (▲) and *Monacha (Monacha)* cf. *compingtae* (▼).

##### Distribution.

This species is here recorded for the first time for Lebanon. It seems to be restricted to higher altitudes up to the alpine region of the highest mountains in the Eastmediterranean.

##### Remarks.

The coordinates of the type locality of *Monacha bari* given by Forcart are erroneous, in fact they should be approximately at 33.3088°N, 35.7596°E. After careful examination of the paratypes of *Monacha bari* from the collection of Forcart (housed in the Natural History Museum in Basel, Switzerland), the recently collected specimens from Lebanon are here identified with this species. It is not possible to find any shell character that could justify a separation of the populations on specific level.

#### Monacha (Monacha) cf. compingtae

(Pallary, 1929)

Fig. 17

Theba compingtae Pallary, Mémoires présentés a l’Institut d’Ègypte 12: 7, pl. 1, figs 4–6. 1929
Monacha cf. *compingtae*, Boessneck, Zoology in the Middle East, 54: figs 5a, 42, 48. 2011

##### Specimens examined.

Zgharta (Prov. North Lebanon), dry ruderal vegetation with calcareous stones in side valley of Nahr Abou Ali above the village, 34.3922°N, 35.8929°E, 80 m alt., leg. U. Boessneck et al.; Hadchit (Prov. North Lebanon), slopes of the Nahr Quadicha, open calcareous fields, locally with loose vegetation und shrubs, partly ruderally impacted, 34.2459°N, 35.9881°E, 1000–1250 m alt., leg. U. Boessneck et al.; Aammiq (Prov. Bekaa), rocky open fields with loose vegetation at the edge of a wetland, ruderally impacted, 33.7284°N, 35.7858°E, 870 m alt., leg. U. Boessneck et al.; Afqa (Prov. Mount Lebanon), open calcareous fields with loose vegetation on single shrubs near the cave, locally wet, 34.0671°N, 35.8291°E, 1160 m alt., leg. U. Boessneck et al.

This *Monacha* species was identified by Hausdorf (in Boessneck 2011) as *Monacha (Monacha)* cf. *compingtae* (Pallary, 1929). We were able to check the specimen figured by Boessneck (2011, Fig. 5a) from Aammiq in order to compare it to the species we finally identified as *Monacha bari*. Both species differ by the possession of a white subsutural spiral band in *Monacha* cf. *compingtae* (not seen in any specimen of *Monacha bari*), and the teleoconch sculpture, which is almost malleate in *Monacha* cf. *compingtae*, while *Monacha bari* shows axial riblets combined with spiral threads.

*Monacha compingtae* was described from Tartus and Safita, both localities from the coastal area in southern Syria. Later, Pallary (1939) extended the distribution area of *Monacha compingtae* with more records from Syria (“kilomètre 139 de la route de Lattaquié à Alep” = probably somewhere NE of Idlib), “Koubba Cheikh Mokbel”, “entre Lattaquié et Kerdaha” and “Slenfé (Nahib Younès” = Slinfah 35.583°N, 36.”°E)). Hausdorf (2000: 84) reported this species from the Hatay area in southern Turkey, which constitutes the northern part of the eastmediterranean mountain ridge. In fact, this species is conchologically very similar to *Monacha compingtae*, the two white spiral bands are already mentioned by Pallary in the original description. However, he also mentions a transversal striation on the shell, which is not seen on the specimen from Aammiq. It might well be that this taxon from Lebanon represents another yet undescribed species.

## Supplementary Material

XML Treatment for Monacha (Monacha) syriaca

XML Treatment for Monacha (Monacha) nummus

XML Treatment for Monacha (Monacha) obstructa

XML Treatment for Monacha (Monacha) crispulata

XML Treatment for Monacha (Monacha) solitudinis

XML Treatment for Monacha (Monacha) bari

XML Treatment for Monacha (Monacha) cf. compingtae
